# Simultaneous Use of Endobronchial and Endoscopic Ultrasound Guidance as Primary Tools in the Diagnosis of Malignant Pleural Mesothelioma

**DOI:** 10.7759/cureus.32110

**Published:** 2022-12-01

**Authors:** Babith Mankidy, Jordan Sparkman, Shreyes Boddu, Quillann Huang, Munish Sharma

**Affiliations:** 1 Pulmonary Critical Care, Baylor College of Medicine, Houston, USA; 2 Gastroenterology, Baylor College of Medicine, Houston, USA; 3 Pulmonary and Critical Care Medicine, Baylor Scott & White Medical Center — Temple, Temple, USA; 4 Oncology, Baylor College of Medicine, Houston, USA; 5 Pulmonary Critical Care, Baylor Scott & White Medical Center — Temple, Temple, USA

**Keywords:** pleural effusion, endoscopic ultrasound, endobronchial ultrasound, pleural biopsy, malignant pleural mesothelioma

## Abstract

Malignant pleural mesothelioma (MPM) is related to exposure to asbestos. It is insidious in nature and is generally diagnosed at an advanced stage. MPM is aggressive and portends a poor prognosis. Definitive diagnosis is usually established by obtaining pathological samples of the pleura by medical or surgical thoracoscopy. However, these procedures are invasive and carry a risk of seeding of biopsy sites with tumors. We herein report an infrequently encountered case of simultaneous use of endobronchial ultrasound and endoscopic ultrasound-guided biopsy of malignant pleural mesothelioma in a 48-year-old female patient.

## Introduction

Mesothelioma is a neoplasm that arises insidiously from mesothelial surfaces like the pleura, pericardium, peritoneum, and tunica vaginalis. It is not a frequently encountered entity with an annual estimate of around 3300 cases per year in the United States [1]. Approximately 82% of cases of mesothelioma are pleural in origin and are referred to as malignant pleural mesothelioma (MPM) [2]. MPM is related to exposure to asbestos and is an aggressive disease with a very grim prognosis despite treatment. Accurate diagnosis of MPM cannot always be obtained by modalities such as thoracentesis and pleural fluid cytology, or by thoracentesis with closed pleural biopsy. Video-assisted thoracoscopic (VATS) biopsy or open thoracotomy are more accurate diagnostic modalities. However, they are invasive in nature and carry the risk of seeding the biopsy site with tumor cells and recurrence at the chest wall. Here we describe a case of MPM that was diagnosed by the simultaneous use of endobronchial ultrasound guidance (EBUS) and endoscopic ultrasound guidance (EUS), thereby avoiding the more invasive traditional modalities.

## Case presentation

A 48-year-old female patient with a history of hypertension, obesity, congestive heart failure with preserved ejection fraction, and 30 pack-year cigarette smoking presented to the pulmonary clinic due to worsening dyspnea on exercise (DOE) for a period of seven months. The patient was at her normal baseline seven months ago when she started noticing DOE. It gradually got worse, to the point where she was unable to go grocery shopping and required an electronic scooter. She also reported intermittent dry cough for two months. The patient did not have any fever, chills, night sweats, chest pain, wheezing, or hemoptysis. There was no history of lung diseases in the family. She did not have any history of autoimmune or connective tissue diseases. There was no occupational exposure to dust or toxic inhalation. She did not have pets at home. Her vital signs revealed blood pressure 146/83 mm Hg, heart rate 75/minute, regular, respiratory rate 18/minute, afebrile, and oxygen saturation of 93% at rest on room air. Examination of the respiratory system revealed normal vesicular breath sounds bilaterally without any adventitious sounds. Other systemic examinations were unremarkable.

**Figure 1:**
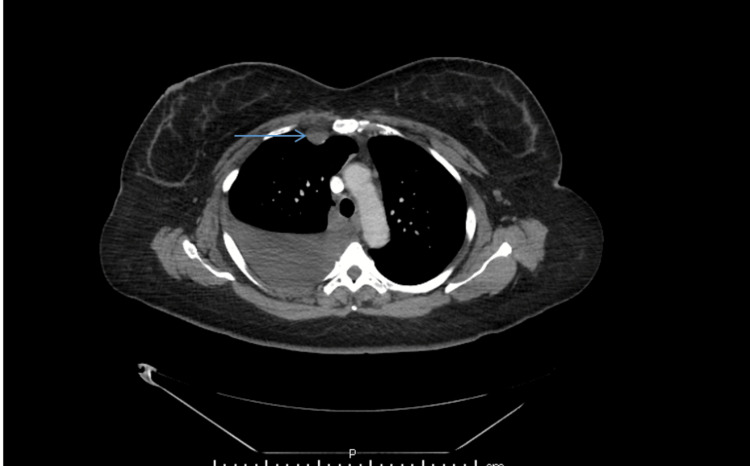
Computed tomography of the chest showing the right internal thoracic lymph node (indicated by blue arrow). The lymph node measured 1.8 x 1.3 cm

**Figure 2:**
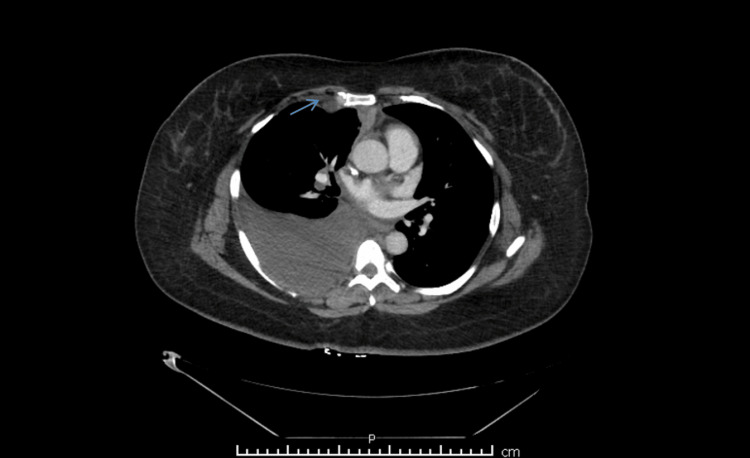
Computed tomography of the chest showing a 1.3 cm-large right internal mammary lymph node (indicated by arrow).

**Figure 3:**
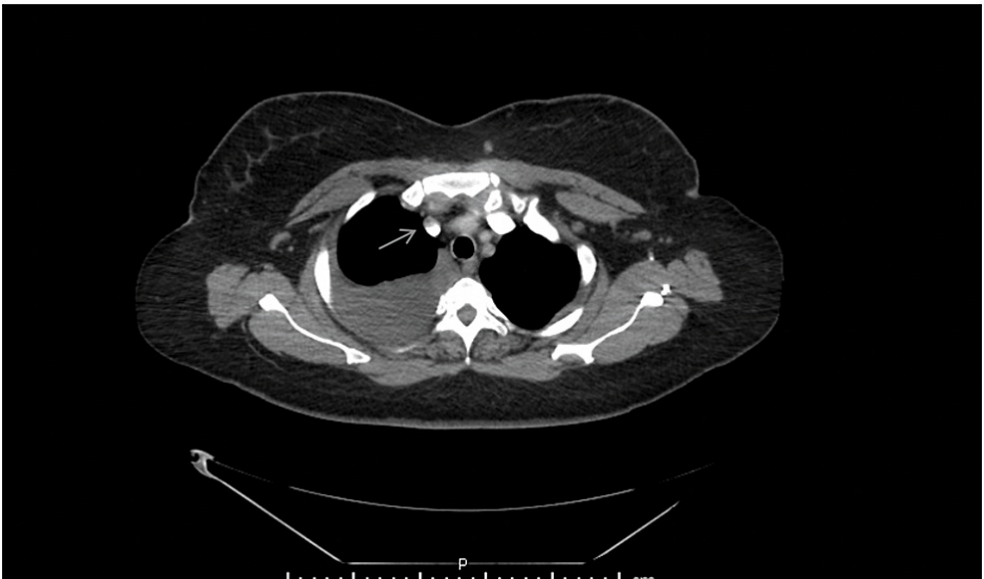
Computed tomography of the chest with intravenous contrast showing a hypoattenuating mass encasing the superior vena cava. The mass is indicated by the arrow.

**Figure 4:**
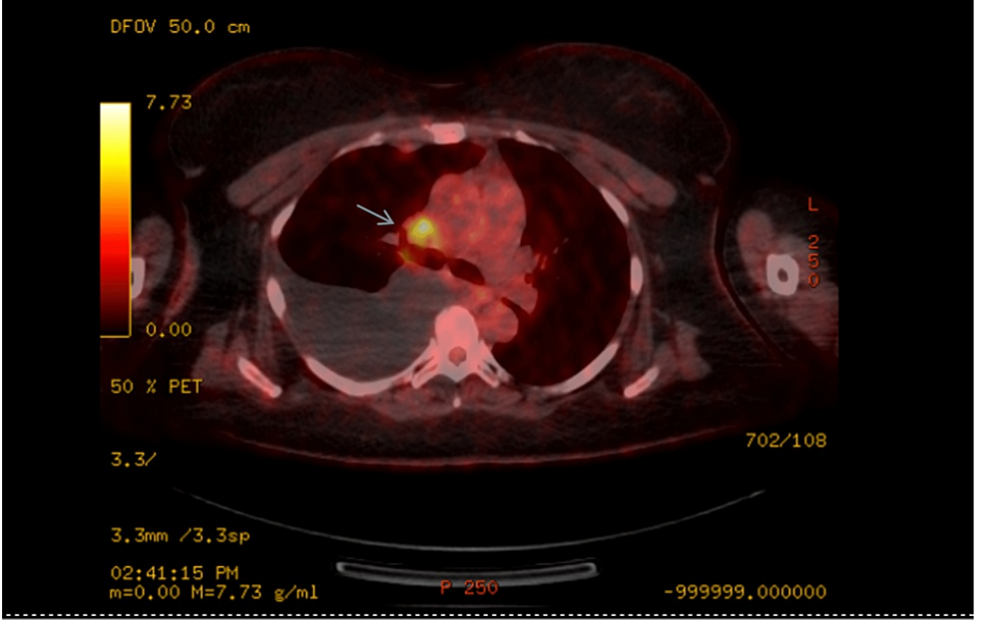
Positron emission tomography/ computed tomography of the chest showing a hypermetabolic mediastinal lymph node indicated by the blue arrow. It also showed moderate volume right pleural effusion.

**Figure 5:**
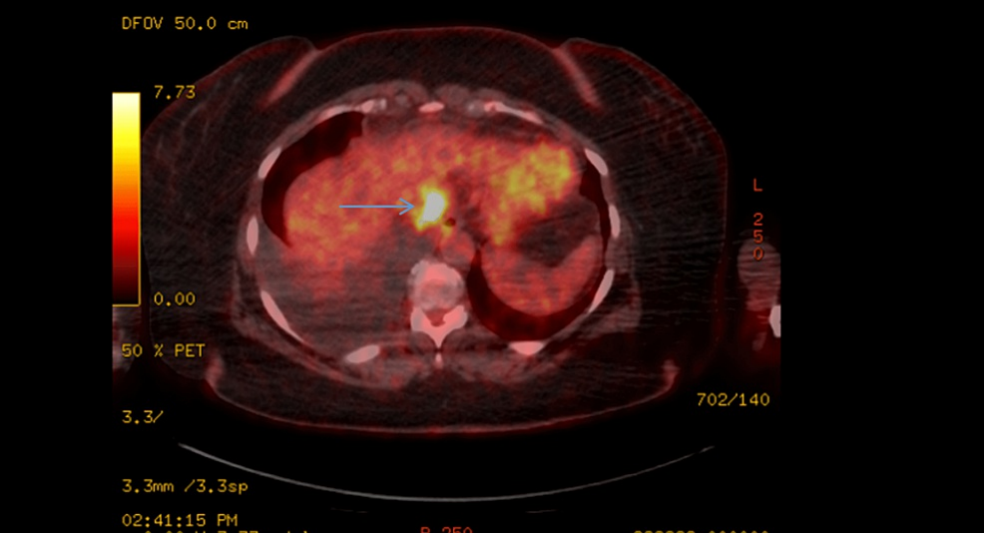
Positron emission tomography/ computed tomography of the abdomen showing a hyper-metabolic para-esophageal lymph node indicated by the arrow.

**Figure 6:**
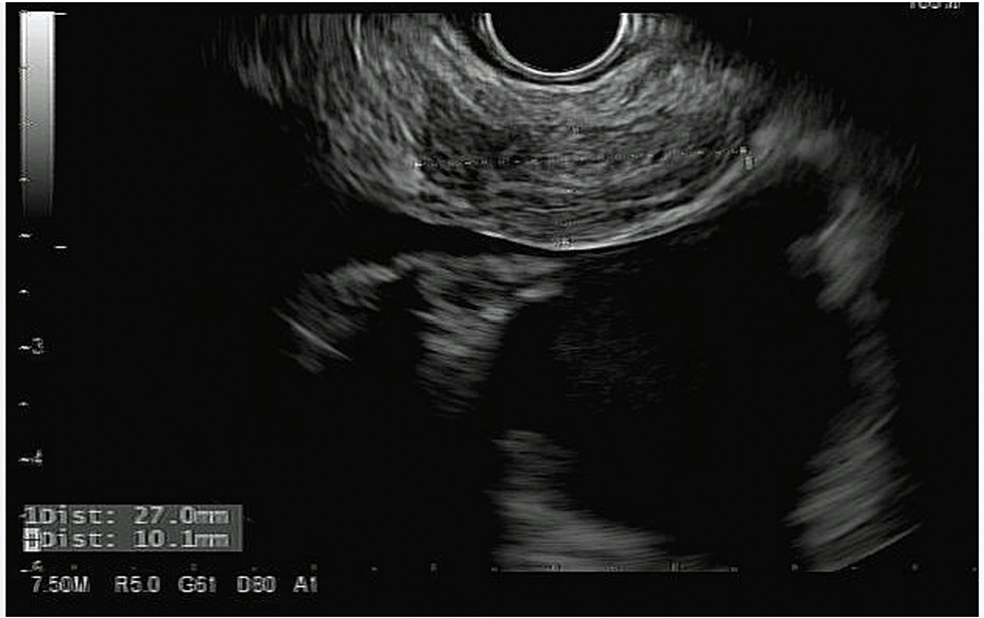
Endoscopic ultrasound image showing an enlarged para-esophageal lymph node.

**Figure 7:**
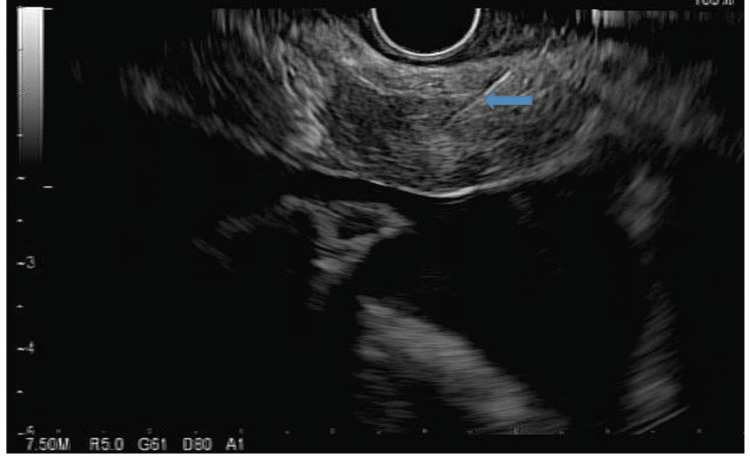
Endoscopic ultrasound image showing needle biopsy of the para-esophageal lymph node (indicated by the arrow).

**Figure 8:**
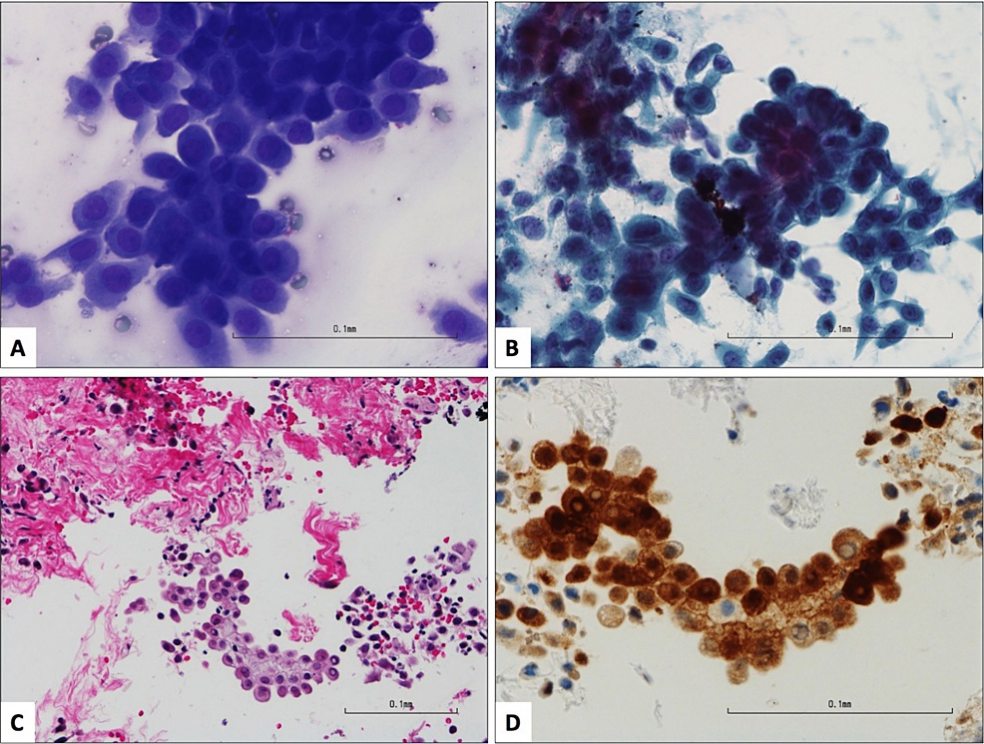
Metastatic malignant mesothelioma illustrated by fine needle aspiration (FNA) and biopsy of the paraesophageal lymph node. FNA smears (A, Diff Quik stain, 400X; B, Papanicolaou stain, 400X) show cohesive clusters of epithelioid cells with monotonous round/ovoid nuclei, fine granular chromatin, small nucleoli, and a moderate amount of cytoplasm. The tumor cells have a hobnailing appearance. The concurrent biopsy (C, hematoxylin, and eosin stain, 200X) displays fibrous tissue and detached clusters of tumor cells with distinct cell borders and hobnailing arrangement. Immunohistochemical stain for calretinin (D, 400X) demonstrates both nuclear and cytoplasmic staining in the tumor cells.

A full pulmonary function test showed decreased forced expiratory volume at 1 sec (FEV1) and forced vital capacity (FVC), normal FEv1/FVC ratio. Total lung capacity (TLC) was decreased while Residual volume (RV)/ TLC ratio was normal. The diffusion capacity of carbon monoxide (DLCO) was mildly reduced (Table 1). The transthoracic echocardiogram showed a left ventricular ejection fraction of 70%, grade I diastolic dysfunction, and normal function and size of the right ventricle. A computed tomography (CT) chest with intravenous contrast showed abnormal hypodense soft tissue in the mediastinum partially encasing and narrowing the inferior segment of the superior vena cava and extending along the right upper lobe pulmonary artery. There were also multiple enlarged right internal thoracic lymph nodes, moderate volume right pleural effusion, and right basilar atelectasis (Figures 1-3). Subsequently, a positron emission tomography (PET) / CT was performed which revealed intensely hypermetabolic right hilar as well as right cardio phrenic and periesophageal lymph node enlargement. There was mildly fluorodeoxyglucose (FDG) avid enlarged right internal mammary lymph nodes versus pleural nodes and FDG uptake along the posterior right 8th rib (Figures 4-5). A thoracentesis with cytology was negative for malignancy. Based on the findings of CT chest and PET/CT, a decision was made to pursue a less invasive diagnostic modality. After an interdisciplinary discussion with Gastroenterology, the patient was subjected to a EUS-guided fine needle biopsy of the para-esophageal lymph nodes near the distal esophagus (Figures 6-7). This was followed in the same setting by EBUS-guided transbronchial needle biopsy of stations 10R and 11 R lymph nodes. Pathological examination showed metastatic malignant mesothelioma (epithelioid type) based on cytomorphologic analysis of samples from para esophageal and station 10 R lymph nodes (Figure 8). Immunohistochemical stains were positive for calretinin, WT 1, D2-40, and cytokeratin 5/6 and negative for thyroid transcription factor 1 (TTF 1), MOC 3, Gata-3, and estrogen receptor (ER). The molecular profile was consistent with malignant pleural mesothelioma, although unfortunately none of these molecular alterations were targetable. After discussion with thoracic surgery, mediastinoscopy and video-assisted thoracic surgery were not deemed necessary as these procedures would not change management. Palliative systemic chemotherapy was planned for this patient with metastatic epithelioid mesothelioma.

**Table 1:** Pulmonary function test FVC: forced vital capacity; FEV1: forced expiratory volume in 1st second; SVC: slow vital capacity; IC: inspiratory capacity; ERV: expiratory reserve volume; TLC: total lung capacity; FRC: functional residual capacity; RV: residual volume; DLCO: diffusion capacity of the lung for carbon monoxide; DLCOadjHb: diffusion capacity of the lung for carbon monoxide adjusted for hemoglobin; VA: alveolar volume

	Actual	Lower limit of normal (LLN)	Z score	Percentage predicted
FVC (liter)	1.89	2.39	-2.84	60
FEV1 (liter)	1.61	1.91	-2.43	64
FEV1/FVC (%)	85.24	70.41	+0.72	105
SVC (liter)	1.96	2.39	-2.67	63
IC (liter)	1.82			59
ERV (liter)	0.14			326
FEV1/SVC (%)	82.24			101
TLC (liter)	3.92	4.40	-3.26	75
FRC (liter)	2.10	0.95	-0.01	99
RV (liter)	1.96	0.96	+0.34	107
RV/TLC (%)	49.99	20.12	+2.18	145
DLCO (ml/min/mmHg)	16.19	16.70	-1.84	75
DLCOadjHb		16.70		
VA ( Liter)	3.59	4.54	-4.12	69

## Discussion

In a patient with clinical suspicion of MPM and relevant abnormal chest X-ray findings, further evaluation with a contrast-enhanced computed tomography (CECT) of the chest is invariably done. Common findings in chest imaging include unilateral pleural effusion, pleural mass or rind-like pleural thickening, and pleural plaques, or calcifications. As with any case of suspected malignancy, establishing a tissue diagnosis is the primary objective in MPM. Attempts can be made to start the work up with less invasive procedures like thoracentesis and pleural fluid cytology or thoracentesis and a closed pleural biopsy. However, studies suggest that the diagnostic yield of MPM is only 26% with pleural fluid cytology and 39 - 44% with thoracentesis and closed pleural biopsy [3-5]. Medical thoracoscopy is also used commonly for the diagnosis of MPM. Studies performed in the late 1980s reported a diagnostic yield of around 60-75% [6,7] while some later data have reported up to 90% diagnostic yield [3]. Data from small case series have reported around 85% and 90% diagnostic yield of MPM with pleuroscopy and biopsy using insulated-tip diathermic knife and cryoprobes respectively [8,9]. However, further data are required before such novel techniques become the universal standard of care. Thus, the gold standard for the diagnosis of MPM has historically been surgical biopsy of the pleura. VATS or open thoracotomy biopsy of pleura are two different approaches. A surgical biopsy can achieve diagnosis in up to 98% of cases [3] but is associated with complications such as subsequent seeding of tumor into the chest wall (10% of cases), pulmonary atelectasis, respiratory failure, and prolonged air leak, amongst others [5,10]. It is also difficult to perform a surgical biopsy on an unstable patient or those with single-lung collapse and fused lung.

MPM per se does not give rise to endobronchial lesions. Thus, the use of flexible fiber-optic bronchoscopy is not routinely utilized in the diagnosis of MPM. EBUS-guided biopsies can be utilized in cases of mediastinal pleural lesions and hilar or mediastinal lymphadenopathy. EBUS-guided biopsies can also help stage the disease if MPM is diagnosed. Endoscopic ultrasound (EUS) can be utilized to enter the esophagus and biopsy paraesophageal lymph nodes under direct ultrasound guidance. A EUS-guided biopsy is reported to be even better tolerated than EBUS-guided biopsies in terms of causing fewer respiratory symptoms such as cough and overall respiratory decompensation [11]. Piro et al. reported that as of January 2021, there were only nine reported cases where EBUS was coupled with EUS to obtain tissue diagnosis of pleural malignancy, whereas two other cases of MPM were diagnosed with the use of EUS-guided biopsy alone [11,12-16].

In our case, after the initial pleural fluid examination was found to be unrevealing, we proceeded with EUS and EBUS-guided fine needle biopsy of the FDG avid mediastinal lymph nodes and obtained a definitive diagnosis. After obtaining the final diagnosis, palliative systemic chemotherapy was the treatment plan. The mainstay of treatment for our patient was platinum-based chemotherapy with cisplatin and pemetrexed. The addition of bevacizumab can be considered in such scenarios based on the benefit shown in the Mesothelioma Avastin Cisplatin Pemetrexed Study (MAPS) [17] although the FDA has not yet approved bevacizumab for this indication. For our patient, treatment with cisplatin, pemetrexed, and bevacizumab was initiated.

## Conclusions

The simultaneous use of EBUS and EUS as primary tools for the diagnosis of MPM is not commonly reported or discussed in the prevailing literature. We should recognize the use of EBUS or EUS as an extra tool in the armamentarium of a clinician when dealing with a possible case of MPM with mediastinal adenopathy. In selected cases, these procedures can provide samples for pathological diagnosis and staging in a rather less invasive manner than conventional techniques. This case also emphasizes the importance of care coordination and utilization of expertise between different sub-specialties in medicine to provide comprehensive patient care. 

## References

[1] Teta MJ, Mink PJ, Lau E, Sceurman BK, Foster ED (2008). US mesothelioma patterns 1973-2002: indicators of change and insights into background rates. Eur J Cancer Prev.

[2] (2022). Centers for Disease Control and Prevention. Incidence of Malignant Mesothelioma, 1999-2018. USCS Data Brief, no. 27. Atlanta, GA: Centers for Disease Control and Prevention, US Department of Health and Human Services. https://www.cdc.gov/cancer/uscs/about/data-briefs/no27-incidence-malignant-mesothelioma-1999-2018.htm.

[3] Boutin C, Rey F (1993). Thoracoscopy in pleural malignant mesothelioma: a prospective study of 188 consecutive patients. Part 1: Diagnosis. Cancer.

[4] Campbell NP, Kindler HL (2011). Update on malignant pleural mesothelioma. Semin Respir Crit Care Med.

[5] Kang B, Kim MA, Lee BY (2013). Malignant pleural mesothelioma diagnosed by endobronchial ultrasound-guided transbronchial needle aspiration. Tuberc Respir Dis (Seoul).

[6] Page RD, Jeffrey RR, Donnelly RJ (1989). Thoracoscopy: a review of 121 consecutive surgical procedures. Ann Thorac Surg.

[7] Davidson AC, George RJ, Sheldon CD, Sinha G, Corrin B, Geddes DM (1988). Thoracoscopy: assessment of a physician service and comparison of a flexible bronchoscope used as a thoracoscope with a rigid thoracoscope. Thorax.

[8] Sasada S, Kawahara K, Kusunoki Y (2009). A new electrocautery pleural biopsy technique using an insulated-tip diathermic knife during semirigid pleuroscopy. Surg Endosc.

[9] Thomas R, Karunarathne S, Jennings B, Morey S, Chai SM, Lee YC, Phillips MJ (2015). Pleuroscopic cryoprobe biopsies of the pleura: a feasibility and safety study. Respirology.

[10] Bydder S, Phillips M, Joseph DJ, Cameron F, Spry NA, DeMelker Y, Musk AW (2004). A randomised trial of single-dose radiotherapy to prevent procedure tract metastasis by malignant mesothelioma. Br J Cancer.

[11] Piro R, Fontana M, Livrieri F (2021). Pleural mesothelioma: When echo-endoscopy (EUS-B-FNA) leads to diagnosis in a minimally invasive way. Thorac Cancer.

[12] Lococo F, Rossi G, Agostini L (2014). "Dry" pleural mesothelioma successfully diagnosed on endobronchial ultrasound (EBUS)-guided transbronchial needle aspiration (TBNA). Intern Med.

[13] Gaspard D, Raja H, Arya R, Abouzgheib W, Boujaoude Z (2015). A case report on the diagnosis of a rare pleural tumor with endobronchial ultrasound: breaking new boundaries. Medicine (Baltimore).

[14] Guinde J, Laroumagne S, Kaspi E, Martinez S, Tazi-Mezalek R, Astoul P, Dutau H (2015). Endobronchial ultrasound in the diagnosis of malignant pleural mesothelioma [article in French]. Rev Mal Respir.

[15] Ghigna MR, Crutu A, Florea V, Soummer-Feulliet S, Baldeyrou P (2016). The role of endobronchial ultrasound-guided fine needle aspiration in the diagnosis of pleural mesothelioma. Cytopathology.

[16] Donghi SM, Prisciandaro E, Sedda G, Guarize J, Spaggiari L (2019). When less is more: EBUS-TBNA for the diagnosis of pleural lesions. Innovations (Phila).

[17] Zalcman G, Mazieres J, Margery J (2016). Bevacizumab for newly diagnosed pleural mesothelioma in the Mesothelioma Avastin Cisplatin Pemetrexed Study (MAPS): a randomised, controlled, open-label, phase 3 trial. Lancet.

